# Multifaceted roles of CARM1 beyond histone arginine methylation

**DOI:** 10.1038/s12276-025-01561-7

**Published:** 2025-10-29

**Authors:** Yena Cho, Yong Kee Kim

**Affiliations:** 1 Muscle Physiome Research Center and Research Institute of Pharmaceutical Sciences, Sookmyung Women’s University, Seoul, Republic of Korea; 2 College of Pharmacy, Sookmyung Women’s University, Seoul, Republic of Korea

**Keywords:** Methylation, Cell signalling

## Abstract

Coactivator-associated arginine methyltransferase 1 (CARM1), first identified in 1999, has been studied primarily for its nuclear role in epigenetic regulation through histone methylation. Subsequent research has expanded the substrate repertoire to include nonhistone proteins, thus uncovering broader functions in maintaining cellular homeostasis by regulating transcription, RNA processing, metabolism and organelle dynamics. More recently, CARM1 was shown to exert scaffolding functions independent of its catalytic activity, thereby orchestrating key signaling events involved in transcriptional activation, replication stress response and cell cycle control. These findings highlight the multifaceted roles of CARM1 in nuclear and cytoplasmic compartments. Despite substantial progress in the development of selective small-molecule inhibitors, their inability to target noncatalytic functions has limited their therapeutic potential. Consequently, novel strategies, such as proteolysis-targeting chimeras, are being explored to degrade the entire CARM1 protein, thereby abolishing its enzymatic and scaffolding functions. Here this review outlines the evolving functional landscape of CARM1, from its roles as a transcriptional coactivator to a multifunctional regulator of cellular homeostasis, with an emphasis on its enzyme-independent functions, thereby providing novel insights for next-generation therapeutic strategies.

## CARM1’s multifaceted roles in cellular homeostasis explored

Arginine methylation is a key process in cells, affecting many functions such as gene expression and DNA repair. This study focuses on CARM1, an enzyme involved in this process. Researchers explored the roles of CARM1 beyond its known nuclear functions. They used various experiments, including studies on mice, to understand how CARM1 works in different parts of the cell. CARM1 was initially known for modifying histones to regulate genes. However, it also affects other proteins outside the nucleus, influencing processes such as metabolism and cell structure. The study found that CARM1 can act without its enzyme activity, serving as a scaffold to support other cellular functions. The research highlights the potential of CARM1 as a target for treating diseases such as cancer. By developing inhibitors and new technologies such as proteolysis-targeting chimeras, scientists aim to block both its enzymatic and nonenzymatic roles.

This summary was initially drafted using artificial intelligence, then revised and fact-checked by the author.

## Introduction

**Fig. 1:**
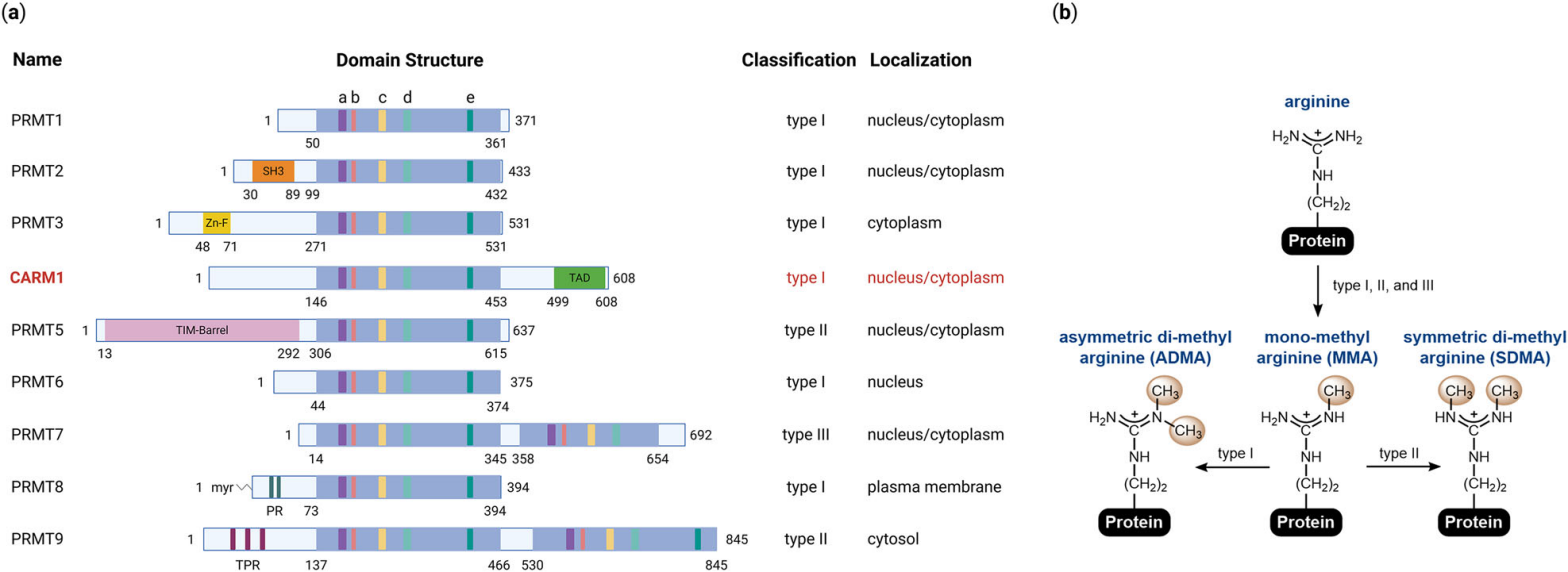
Overview of PRMTs. **a** The mammalian PRMT family. Nine PRMTs have been identified in mammals. These enzymes share unique signature motifs with high sequence similarity, including: motif I: VLD/EVGXGXG (a); post-I: V/IXG/AXD/E (b); motif II: F/I/VDI/L/K (c); motif III: LR/KXXG (d); and the THW loop (e). Enzymatic types (type I, II or III) and subcellular localizations of each PRMT are indicated. **b** Types of arginine methylation. The arginine residue contains two equivalent nitrogen atoms in its guanidino group. All three PRMT types (I, II and III) can catalyze MMA formation. Type I PRMTs (PRMT1, PRMT2, PRMT3, CARM1, PRMT6 and PRMT8) further catalyze the formation of ADMA, whereas type II PRMTs (PRMT5 and PRMT9) generate SDMA. Type III PRMT (PRMT7) exclusively produces MMA.

Arginine methylation, first identified in 1967^1^, is a crucial post-translational modification (PTM) that regulates a broad spectrum of cellular processes, including transcription, RNA processing, DNA damage response and signal transduction^2–4^. This modification is catalyzed by nine protein arginine methyltransferases (PRMTs), all of which share an *S*-adenosylmethionine (AdoMet)-binding domain characterized by conserved sequence motifs (I, post-I, II and III)^5,6^ (Fig. 1a). Following the discovery of PRMT1 in 1996^7^, additional members of the PRMT family were identified over the next decade: PRMT2 and PRMT3 in 1998^8,9^, CARM1 and PRMT5 in 1999^10,11^, PRMT6 in 2002^12^, PRMT7 in 2004^13^ and PRMT8 and PRMT9 in 2005^14^. These enzymes transfer a methyl group from AdoMet to the guanidino group of arginine residues on substrate proteins^15^. Based on the type of methylarginine they generate, PRMTs are classified into three subtypes: type I (PRMT1, 2, 3, 4, 6 and 8) catalyze the formation of monomethylarginine (MMA) and asymmetric dimethylarginine (ADMA), type II (PRMT5 and 9) produce MMA and symmetric dimethylarginine (SDMA), and type III (PRMT7) exclusively generates MMA^16^ (Fig. 1b).

Among these enzymes, coactivator-associated arginine methyltransferase 1 (CARM1; also known as PRMT4) functions as a transcriptional coactivator with histone methyltransferase activity^10^. It was originally characterized by its ability to dimethylate histone H3 at arginines 17 and 26 (H3R17me2a and H3R26me2a), contributing to chromatin remodeling and transcriptional activation^17–19^. These findings indicated that CARM1 is a key nuclear epigenetic regulator. However, over the past two decades, the functional repertoire of CARM1 has expanded considerably. Beyond its nuclear role in histone modification, CARM1 has been shown to methylate a wide range of nonhistone substrates—including RNA-binding proteins (such as PABP1^20^, p54nrb^21^ and SAP49^22^), transcriptional regulators (such as MED12^23^ and SRC-3^24^), metabolic enzymes (such as pyruvate kinase M2 (PKM2)^25,26^, malate dehydrogenase 1 (MDH1)^27^ and GAPDH^28^) and others (such as dynamin-related protein 1 (DRP1)^4^ and PI3KC2α^29^). These discoveries have highlighted the involvement of CARM1 in cytoplasmic processes such as RNA processing, metabolism, cytoskeletal organization, autophagy and organelle dynamics, thus challenging the earlier notion that arginine methylation occurs predominantly in the nucleus^30^. Increasing evidence has shown that CARM1 plays a noncatalytic role in cells. Although genetic and biochemical studies have underscored the importance of its enzymatic activity^31^, recent findings indicate that CARM1 may also function as a scaffolding or structural component in various signaling contexts^32–35^, further broadening its biological relevance.

Notably, CARM1 has emerged as a promising therapeutic target for multiple diseases, including cancer^36,37^, metabolic disorders^38^ and aging^39,40^. Since aberrant overexpression or hyperactivation of CARM1 has been implicated in tumor progression, pharmacological inhibition of its activity has shown efficacy in preclinical models^41–46^. This has encouraged the development of selective small-molecule inhibitors (such as EZM2302^43^ and TP-064^47^) and proteolysis-targeting chimeras (PROTACs) that degrade CARM1 with high specificity^48^. In this Review, we provide a comprehensive overview of CARM1 biology by tracing its evolution from a transcriptional coactivator to a multifunctional regulator of cellular homeostasis. We focus on emerging evidence that extends beyond its canonical nuclear and catalytic functions, emphasizing nonnuclear and enzyme-independent mechanisms that may provide novel opportunities for therapeutic interventions.

## Short history of CARM1

**Fig. 2:**
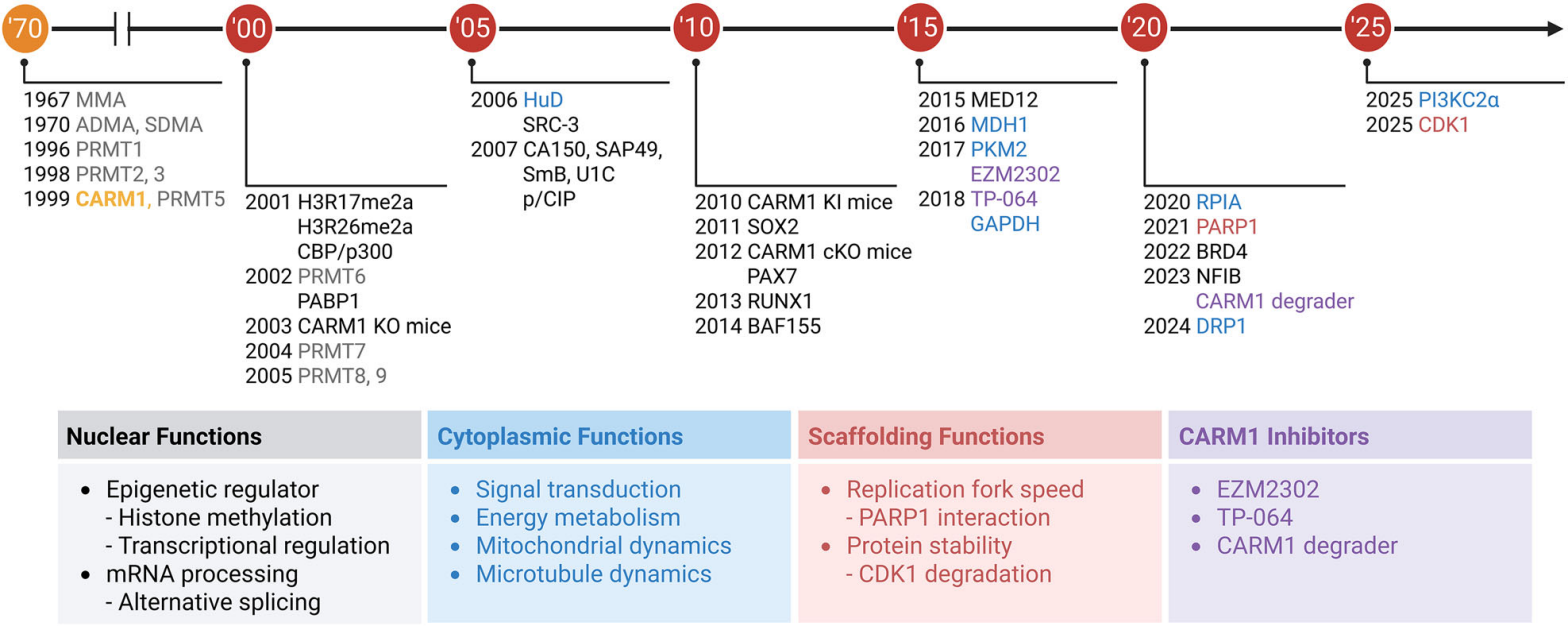
A brief history of functional and pharmacological studies of CARM1. The discovery of methylated arginine residues in 1967 initiated the study of PRMTs, although the first enzyme, PRMT1, was not cloned until 1996. Since then, nine members of the PRMT family have been identified. Among these, CARM1 was first identified in 1999 using yeast two-hybrid screening. Initially characterized as an epigenetic regulator that modulates transcription through histone methylation, CARM1 possesses diverse cytoplasmic functions and scaffolding roles that contribute to cellular homeostasis. In recent years, the development of selective CARM1 inhibitors has enabled a more detailed investigation of their biological functions and therapeutic potential.

CARM1 was first identified in 1999 as a histone methyltransferase that enhances transcriptional activation by nuclear hormone receptors in cooperation with SRC-1, GRIP1/TIF2 and p/CIP^10^ (Fig. 2). Various genetically engineered mouse models have been developed to elucidate physiological relevance. In 2003, a *Carm1* knockout (KO) mouse model revealed partial embryonic lethality at late gestation (E18.5–E19.5), with surviving embryos exhibiting growth retardation and perinatal death due to respiratory failure^49,50^. These mice also displayed defects in T cell development^51^, adipogenesis^52^ and chondrogenesis^53^, underscoring the essential role of CARM1 in development. To determine the contribution of enzymatic activity, a catalytically inactive *Carm1* knock-in (KI) mouse model was created in 2010. These enzyme-dead KI mice closely recapitulated the phenotype observed in full-KO models^31^, revealing that the biological functions of CARM1 largely depend on its methyltransferase activity. Recently, conditional knockout (cKO) mouse models were used to explore the tissue-specific roles of CARM1. In 2012, muscle-specific deletion of *Carm1* using *Pax7-Cre* revealed that CARM1-mediated methylation of PAX7 is critical for the asymmetric division of satellite cells and skeletal muscle regeneration^54^. A subsequent study using human skeletal actin promoter-driven *Cre* showed that CARM1 modulates AMPK signaling and autophagy, thereby regulating muscle mass and atrophy^55^. In the male germline, *Carm1* deletion through *Stra8-Cre* uncovered its role in late-stage spermatid maturation, where it antagonizes the transcriptional activity of the p300/ACT/CREMτ complex, although it is dispensable for spermatocyte development^56^. In 2019, a *Cre*-inducible *Carm1* overexpression model was developed to evaluate its oncogenic potential. This model revealed that although CARM1 overexpression alone did not initiate tumorigenesis, it significantly enhanced tumor progression in cooperation with oncogenic drivers such as mutant ERBB2/Neu^57^. Collectively, these genetic models have provided critical insights into the diverse physiological and pathological functions of CARM1, from tissue-specific differentiation to tumor progression, and have underscored its therapeutic relevance in both developmental disorders and cancers.

## The molecular functions of CARM1

Although CARM1 was initially characterized as a transcriptional coactivator that modifies histone H3, subsequent studies revealed a remarkably broad substrate repertoire that spans diverse cellular compartments and biological functions. Unlike other PRMTs that preferentially target glycine–arginine-rich motifs^58^, CARM1 displays unique substrate specificity, favoring arginine residues within proline-, glycine- and methionine-rich regions, which are often located in proline-rich domains^20,22,59^. This catalytic activity enables CARM1 to fine-tune cellular physiology by regulating protein–protein and protein–RNA interactions, enzymatic activity, protein stability and subcellular localization through site-specific methylation. Notably, the functional outcome of CARM1-mediated methylation is highly context dependent. In the nucleus, CARM1 modifies histone and nonhistone proteins to modulate chromatin accessibility and transcriptional activation^19,60^, whereas in the cytoplasm, it regulates mitochondrial fission, glycolysis and anabolic metabolism^4,26–28^. These observations highlighted the importance of CARM1 as a multifunctional enzyme that integrates PTM signals to coordinate transcriptional and metabolic programs. A comprehensive list of CARM1 substrates, their specific methylation sites and their associated cellular functions is summarized in Table 1. This catalog provides insights into how arginine methylation by CARM1 contributes to diverse cellular processes, ranging from gene expression and RNA processing to energy metabolism and organelle dynamics. Understanding this substrate-specific regulation is essential for elucidating the biological impact of CARM1 activity and its potential as a therapeutic target.

**Table 1:** A list of validated CARM1 substrates.

Substrate	CARM1-methylated arginine residues	Function	References
**Nucleus**			
ASXL2	R639, R641	Blocks binding to MLL3	^91^
BAF155	R1064	Switches promoter occupancy from BAF155 to EZH2	^89^
CA150	R28, R30, R41, R48	Enhances interaction with SMN	^22^
CARM1	R550 (only CARM1-FL)	Affects pre-mRNA splicing	^61,82^
CBP	R601, R625	Blocks CREB activation by inhibiting the binding between CBP/p300 and CREB	^112^
	R714, R742, R768, R2151	Increases histone acetyltransferase activity	^83,113^
p300	R580, R604, R651	Blocks CREB activation by inhibiting the binding between CBP/p300 and CREB	^112^
	R754	Induces interaction with BRCA1	^90^
	R2142	Impairs binding to GRIP1 and ACT	^56,114^
Histone H3	R17, R42	Activates transcription	^17–19,115^
	R26	Regulates transcription	^17,18,116,117^
HuR	R217	Affects subcellular localization and stability	^118^
KDM1A (LSD1)	R838	Stabilizes KDM1A by promoting binding to USP7	^119^
MED12	R1782, R1792, R1854, R1859, R1871, R1910, R1994, R2015	Activates estrogen/ERα-induced gene transcription	^64^
	R1862, R1912	Suppresses *CDKN1A* transcription	^23^
	R1899	Promotes interaction with TDRD3	^98^
NFIB	R388	Promotes the transcription of its target genes	^46^
p/CIP (NCOA3)	R849, R854, R1171, R1177, R1188	Impairs association to CBP/p300	^24,120^
p54nrb	R357, R365, R378	Decreases binding to mRNAs containing IRAlus	^21^
PABP1	R455, R460, R506	Unknown; no impact on protein stability and subcellular distribution	^20,121^
PAX7	R10, R13, R22, R37	Increases *Myf5* expression through interaction with MLL complex	^54^
PRMT5	R505	Facilitates homodimerization	^122^
Rb	R775, R787, R798	Facilitates phosphorylation of Rb and inactivates its tumor suppressor activity	^123^
RNA pol II	R1810	Affects the expression of select RNAs and provides a binding site for TDRD3	^124^
RUNX1	R223, R319	Triggers the assembly of a multiprotein repressor complex containing DPF2	^70^
SOX2	R113	Increases SOX2 transcriptional activity	^125^
**Cytoplasm**			
ACSL4	R339	Degrades ACSL4 by promoting binding to RNF25	^84^
DRP1	R403, R634	Promotes mitochondrial recruitment and fission	^4^
GAPDH	R234	Inhibits glycolysis	^28^
HuD	R236	Affects *CDKN1A* mRNA stability	^99^
MDH1	R230	Inhibits dimerization and activity	^27^
PI3KC2α	R175	Increases protein stability	^29^
PKM2	R445, R447, R455	Inhibits InsP_3_R-mediated calcium signaling	^25^
	R445, R447	Enhances tetramerization and activity	^26^
RPIA	R42	Increases activity	^100^

## Subcellular localization and isoform-specific functions of CARM1

**Fig. 3:**
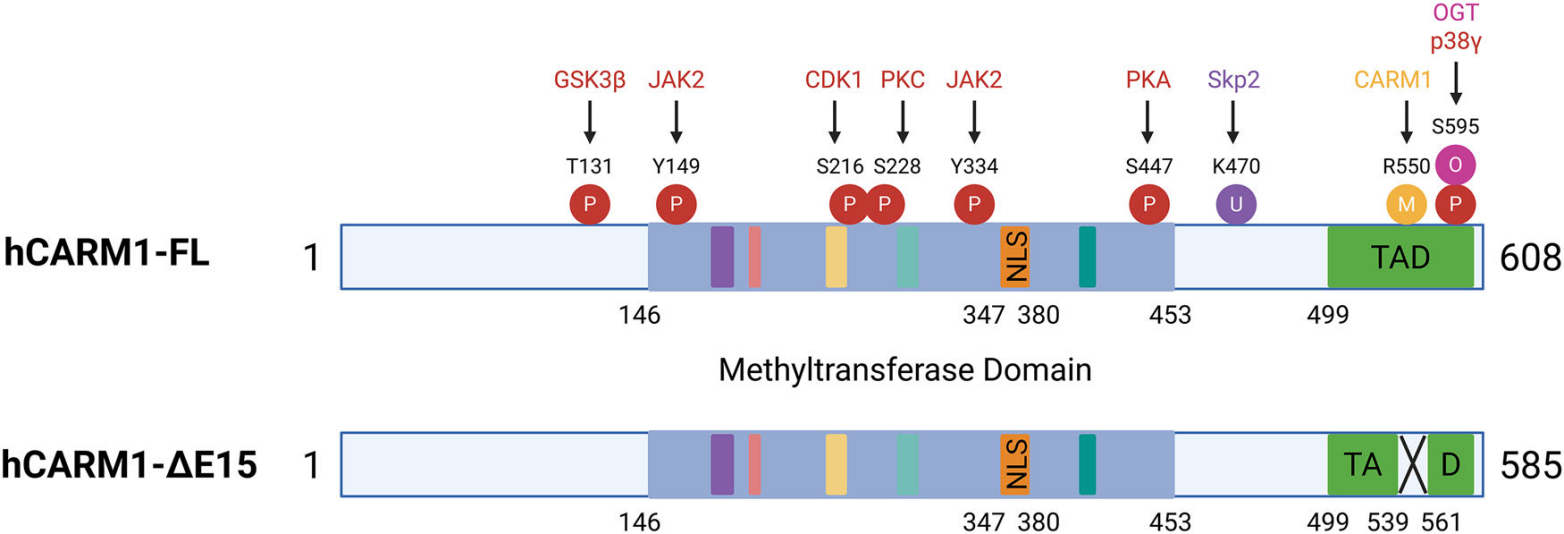
Structural domains and post-translational modifications of CARM1. A schematic representation of full-length human CARM1 (hCARM1-FL) and its alternatively spliced isoform (hCARM1-ΔE15), illustrating domain organization and major PTM sites. The methyltransferase domain, nuclear localization signal (NLS) and transactivation domain (TAD) are shown. Key phosphorylation (P), methylation (M), ubiquitination (U) and O-GlcNAcylation (O) sites, along with their corresponding modifying enzymes (such as GSK3β, CDK1, PKA and Skp2), are indicated. Notably, the ΔE15 isoform lacks exon 15 (amino acids 539–561), resulting in a truncated TAD. All residue numbers correspond to the human CARM1 protein sequence for consistency. CDK1, cyclin-dependent kinase 1; GSK3β, glycogen synthase kinase 3 beta; JAK2, Janus kinase 2; OGT, O-GlcNAc transferase; PKA, protein kinase A; PKC, protein kinase C; Skp2, S-phase kinase-associated protein 2.

CARM1 is frequently overexpressed in various cancers and exists in multiple alternatively spliced isoforms, each of which exhibits distinct biological properties. The two predominant isoforms are the full-length variant (CARM1-FL; 608 amino acids) and a shorter form lacking exon 15 (CARM1-ΔE15; 585 amino acids)^44,61^ (Fig. 3). These isoforms differ in structure and function: although CARM1-FL is associated with tumor-suppressive activity, CARM1-ΔE15 has been linked to oncogenic properties, altered enzymatic behavior and changes in subcellular localization^61,62^. For example, in triple-negative breast cancer, CARM1-ΔE15 is predominantly cytoplasmic, whereas CARM1-FL is largely nuclear^62^. In small cell lung cancer, the ratio of CARM1-FL to CARM1-ΔE15 correlates with differential responses to chemotherapy^63^. These observations indicate that the relative abundance and localization of CARM1 isoforms are critical determinants of the cancer phenotype and therapeutic response, highlighting their potential as biomarkers for precision medicine.

Early studies on the role of CARM1 in cancer have yielded conflicting results. Some reports have implicated CARM1 in promoting tumor growth, invasion and metastasis in breast^42,64,65^, ovarian^66,67^, prostate^68^, hematologic^69,70^ and colorectal cancers^71^. In contrast, other studies have reported tumor-suppressive roles in lung^50^, liver^28^, and pancreatic cancers^27^. These discrepancies may be partially explained by the isoform-specific expression. Notably, CARM1-ΔE15 lacks the automethylation site R550, which is essential for substrate recognition and methyltransferase activity^61^. This structural difference could notably alter the functional output of the enzyme, contributing to its context-dependent behavior across tissue types.

In addition to CARM1-FL and CARM1-ΔE15, other isoforms such as CARM1-v2 (651 amino acids) and CARM1-v3 (573 amino acids), resulting from intron retention events, have been identified in the rat^72^. These variants exhibit tissue-specific expression patterns: CARM1-FL is abundant in the heart and skeletal muscle; v2 is enriched in the liver, brain and testis; and v3 is predominantly found in the adult kidneys, spleen and fetal brain^72^. This diversity further underscores the importance of considering isoform-specific roles in both normal physiology and disease.

Collectively, these findings indicate that the biological functions of CARM1 are highly dependent on its isoform expression and subcellular distribution. Understanding this complexity is essential for the development of targeted therapeutic strategies and for leveraging CARM1 as a prognostic or predictive biomarker for cancer.

## Regulatory mechanism of CARM1

The enzymatic activity and cellular functions of CARM1 are regulated by various PTMs that modulate its localization, stability, substrate specificity and catalytic output (Fig. 3). These modifications, in conjunction with the isoform diversity, define the functional plasticity of CARM1 in both physiological and pathological contexts.

### Phosphorylation

Phosphorylation is a key regulatory mechanism of CARM1. During mitosis, CDK1 phosphorylates CARM1 at serine 216 (S216) in the late G2 phase, leading to the suppression of enzymatic activity and cytoplasmic translocation^35,73^. Once in the cytoplasm, CARM1 undergoes further phosphorylation at S228 by PKC, which disrupts homodimerization and exacerbates enzymatic inhibition^35,74,75^. These events reflect the coordinated cell cycle-dependent repression of CARM1 activity. Conversely, phosphorylation can also enhance CARM1 function. For instance, PKA-mediated phosphorylation at S447 facilitates interaction with ligand-free estrogen receptor alpha (ERα), thereby promoting cAMP-responsive gene expression^33^. In addition, p38γ MAPK phosphorylates CARM1 at S595, enhancing its cytoplasmic accumulation^39,76^. This relocalization shifts the substrate preference by reducing nuclear PAX7 methylation^76^ and promoting cytoplasmic DRP1 methylation^4,39^. In oncogenic contexts such as reactive oxygen species (ROS)-driven signaling or JAK2 V617F-mutant leukemia, tyrosine phosphorylation at Y149 and Y334 by JAK2 enhances both the nuclear localization and substrate specificity of CARM1^77^, indicating that aberrant kinase signaling sustains CARM1 in a constitutively active state.

### O-GlcNAcylation

CARM1 is regulated by O-GlcNAcylation, a nutrient-sensitive PTM that occurs at S595, S598, T601 and T603. Although O-GlcNAcylation did not affect protein stability or localization, it modulated substrate selectivity in response to metabolic stress^78^, revealing that CARM1 acts as a sensor that integrates nutrient availability into transcriptional and post-transcriptional regulation.

### Other modifications

Additional regulatory inputs further diversify function of CARM1. GSK3β-mediated phosphorylation at T131 prevents ubiquitination, thereby stabilizing CARM1 protein levels^79^. Conversely, the Skp2-dependent ubiquitination at K470 under nutrient-rich conditions promotes CARM1 degradation^80,81^. Automethylation at R550, a site absent in the oncogenic ΔE15 isoform, is essential for efficient substrate binding and regulates interactions involved in transcription and RNA splicing^61,82^.

Through these combinatorial PTMs, CARM1 switches between nuclear and cytoplasmic functions, modifies its interaction networks and fine-tunes its substrate repertoire. This regulation is especially critical in cancer, where the role of CARM1 extends beyond transcriptional coactivation to include functions such as cytoplasmic scaffolding, mitochondrial dynamics and immune modulation. CARM1 has been implicated in diverse processes including RNA splicing, biomolecular condensate formation through phase separation, T cell differentiation and NF-κB signaling. Collectively, the extensive regulation of CARM1 by PTMs and alternative splicing highlights its complex functional landscape. These modifications allow for precise spatial and temporal control of its enzymatic activity and substrate interactions, enabling CARM1 to respond dynamically to cellular cues such as cell cycle progression, metabolic status and oncogenic signaling. In this review, we highlight these mechanisms with particular emphasis on emerging nonnuclear roles, which expand our understanding of CARM1 as a multifaceted regulator in health and disease.

## The cellular functions of CARM1

As mentioned previously, CARM1 exerts diverse cellular functions as determined by its substrate specificity, which is modulated by changes in enzymatic activity and subcellular localization through PTMs. Under normal physiological conditions, CARM1 is predominantly localized to the nucleus, where it performs epigenetic functions by methylating histone proteins, transcriptional coregulators and proteins involved in mRNA processing^19,22,60,83^. In contrast, a smaller portion of CARM1 is localized in the cytoplasm, where it contributes to essential processes such as energy metabolism, cytoskeletal organization, signal transduction and maintenance of cellular homeostasis^25,28,29,84^. However, recent studies have shown that under pathophysiological conditions, CARM1 is increasingly translocated to the cytoplasm where it aberrantly methylates nonnuclear proteins, an event implicated in disease pathogenesis^4,39^. This notion is further supported by findings that triple-negative breast cancer frequently overexpresses a cytoplasm-enriched splice variant, CARM1-ΔE15^61^. Accumulating evidence indicates that CARM1 contributes to cellular homeostasis through its enzymatic activity as well as noncatalytic scaffolding functions.

## Nuclear functions: transcriptional regulation

Over the past two decades, CARM1 has been recognized for its pivotal role in transcriptional regulation. CARM1-mediated histone arginine methylation modulates chromatin remodeling and facilitates the assembly of coactivator complexes, often in concert with histone acetylation. Pre-acetylation of histones by CBP/p300 facilitates the recruitment of CARM1 to chromatin, enhances H3R17 methylation and establishes a transcriptionally permissive environment^19,85^. Mechanistically, acetylation of H3K18 potentiates CARM1-mediated H3R17 methylation not by increasing substrate affinity, but by enhancing the catalytic efficiency (*k*_cat_) of the methyltransferase reaction. Structural studies have shown that K18 acetylation neutralizes the positive charge at the +1 position relative to R17, thereby reducing electrostatic interference in the active site and facilitating the essential proton transfer required for catalysis^6^. This electrostatic tuning mechanism ensures efficient H3R17 methylation and exemplifies a specific syntactical feature of the histone code.

**Fig. 4:**
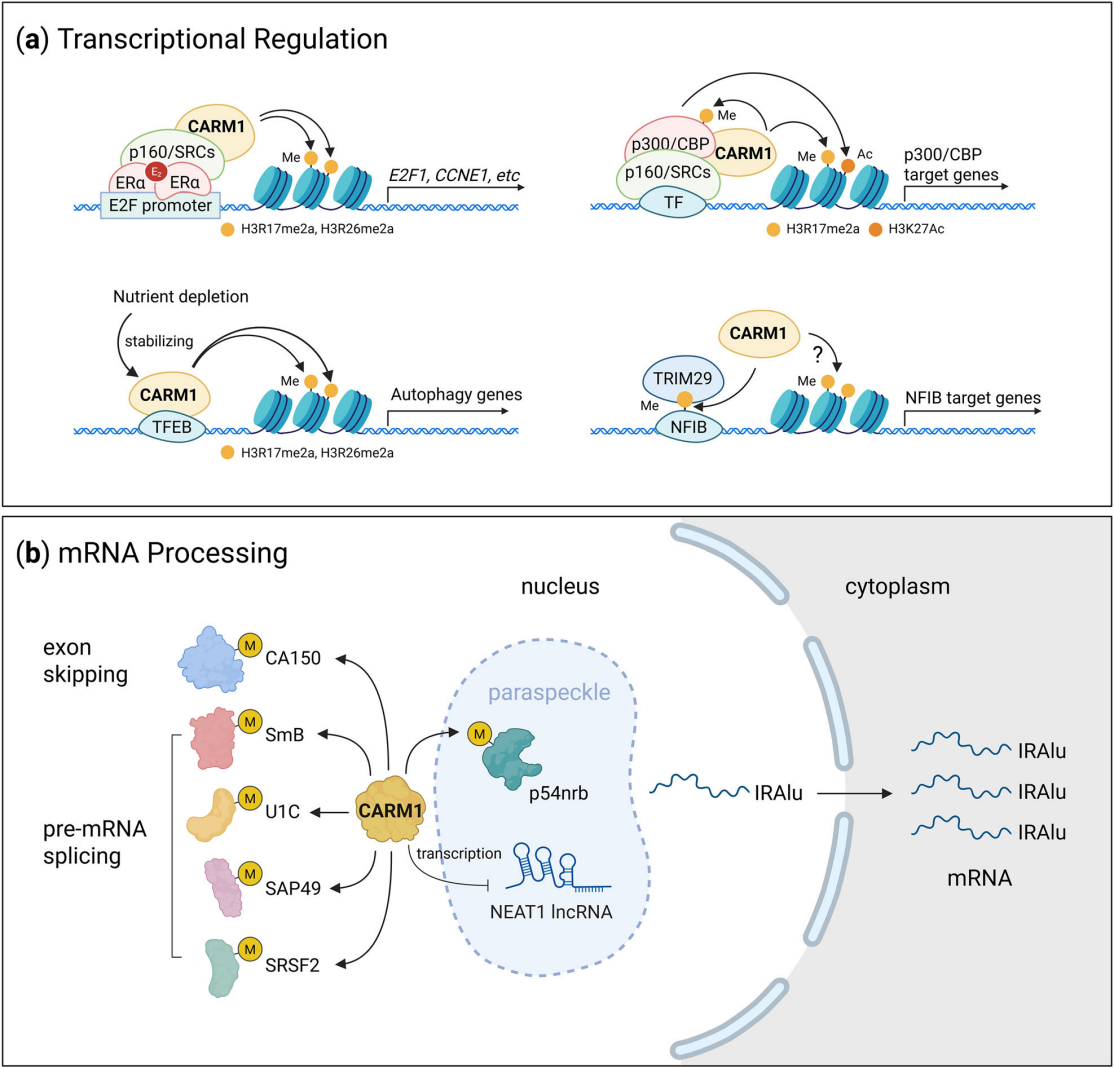
Nuclear functions of CARM1. **a** Transcriptional regulation by CARM1. CARM1 regulates gene expression by methylating both histone and nonhistone proteins involved in the transcriptional machinery. These methylation events, including H3R17me2a and H3R26me2a, often act synergistically with histone acetylation to promote transcriptional activation by facilitating chromatin remodeling and the displacement of corepressors. **b** Regulation of mRNA processing by CARM1. CARM1 controls mRNA maturation through methylation of multiple splicing factors (such as CA150, SmB, U1C, SAP49 and SRSF2), thereby influencing exon skipping and pre-mRNA splicing. In addition, CARM1 regulates nuclear mRNA export by modulating paraspeckle function through two mechanisms: (1) suppressing transcription of the long noncoding RNA NEAT1 and (2) methylating p54nrb, which decreases its binding affinity to IRAlu-containing mRNAs. Under cellular stress, reduced CARM1 levels in paraspeckles lead to nuclear retention of IRAlu-mRNAs. *CCNE1*, cyclin E1 gene; E2F1, E2F transcription factor 1; NFIB, nuclear factor I B; p160/SRCs, steroid receptor coactivators; TF, transcription factor; TRIM29, tripartite motif-containing protein 29.

Beyond its role in general chromatin modulation, CARM1 controls gene-specific transcription in diverse biological contexts. For example, 17β-estradiol (E_2_) induces CARM1 recruitment to the E2F1 promoter, resulting in increased H3R17me2a and transcriptional activation of E2F1 and its downstream targets (*CDC25A*, *CCNA1*, *CCNE1* and *CCNE2*), thereby promoting ERα-positive breast cancer progression^86^ (Fig. 4a). Similarly, CARM1 upregulates *SERPINE1* transcription in the presence of LRRFIP2 variant 3, thereby facilitating gastric cancer cell proliferation^87^. Moreover, CARM1 facilitates TFEB-mediated transcription of autophagy and lysosomal genes through H3R17me2a deposition at their promoters^80,88^, underscoring its key role in autophagy induction in response to nutrient starvation (Fig. 4a).

In addition to histone modifications, CARM1 regulates gene expression through methylation of various nonhistone substrates. For example, it modulates the c-Myc transcriptional network by methylating BAF155, a core subunit of the SWI/SNF chromatin-remodeling complex^89^. In addition, CARM1 promotes BRCA1-mediated transcription of cyclin-dependent kinase inhibitor 1A (p21) (*CDKN1A*) by methylating CBP/p300, thereby stabilizing the interaction between BRCA1 and its coactivators^90^. CARM1-dependent activation of the NFIB–TRIM29 complexes was also shown to drive *PDE1A* transcription^46^ (Fig. 4a). However, CARM1 can also function as a transcriptional repressor in certain contexts. A recent study identified ASXL2, a component of the BAP1 complex, as a novel substrate for CARM1. Methylation of ASXL2 by CARM1 prevents the recruitment of MLL3 to enhancer regions, leading to the repression of MLL3/COMPASS-dependent gene expression^91^. Collectively, these findings indicate that CARM1 can function as both a transcriptional coactivator and corepressor depending on the specific substrates it modifies and the composition of its interacting protein complexes.

## Nuclear functions: mRNA processing

Although CARM1 is best known for its role in transcriptional regulation, it also governs a variety of nuclear processes, including RNA processing, such as pre-mRNA splicing^22,72,82,92,93^, nuclear mRNA export^21^ and the regulation of noncoding RNAs^71,94–96^. The CARM1-v3 isoform regulates 5′ splice site selection in a methylation-independent manner by directly interacting with U1C, a component of the U1 snRNP complex^72^. In contrast, CARM1-FL promotes exon skipping and modulates pre-mRNA splicing through methylation of CA150 and several splicing regulators, including U1C, SmB, SAP49 and SRSF2^22,93^. Through these activities, CARM1 orchestrates context-dependent alternative splicing programs. Furthermore, CARM1 regulates nuclear mRNA retention in paraspeckles—subnuclear structures formed around the long noncoding RNA NEAT1. Specifically, CARM1 represses *NEAT1* transcription, thereby limiting paraspeckle formation. It also methylates p54nrb, reducing its affinity for inverted repeat Alu elements (IRAlus) in mRNAs. This leads to enhanced nuclear export of IRAlus-containing mRNAs and facilitates their translation into the cytoplasm^21,97^ (Fig. 4b). In addition, CARM1 plays a pivotal role in noncoding RNA functions. CARM1-mediated methylation of MED12 facilitates the recruitment of the TDRD3–TOP3B complex, enhancing the interaction of MED12 with enhancer RNAs (eRNAs). This modification also promotes the recruitment of CBP/p300, which, in turn, activates eRNA transcription through H3K27 acetylation. These eRNAs subsequently regulate H3K4me3 enrichment at immunoglobulin switch regions, thereby promoting class switch recombination by recruiting components of DNA damage and repair machinery^98^.

## Cytoplasmic functions: mRNA decay and energy metabolism

**Fig. 5:**
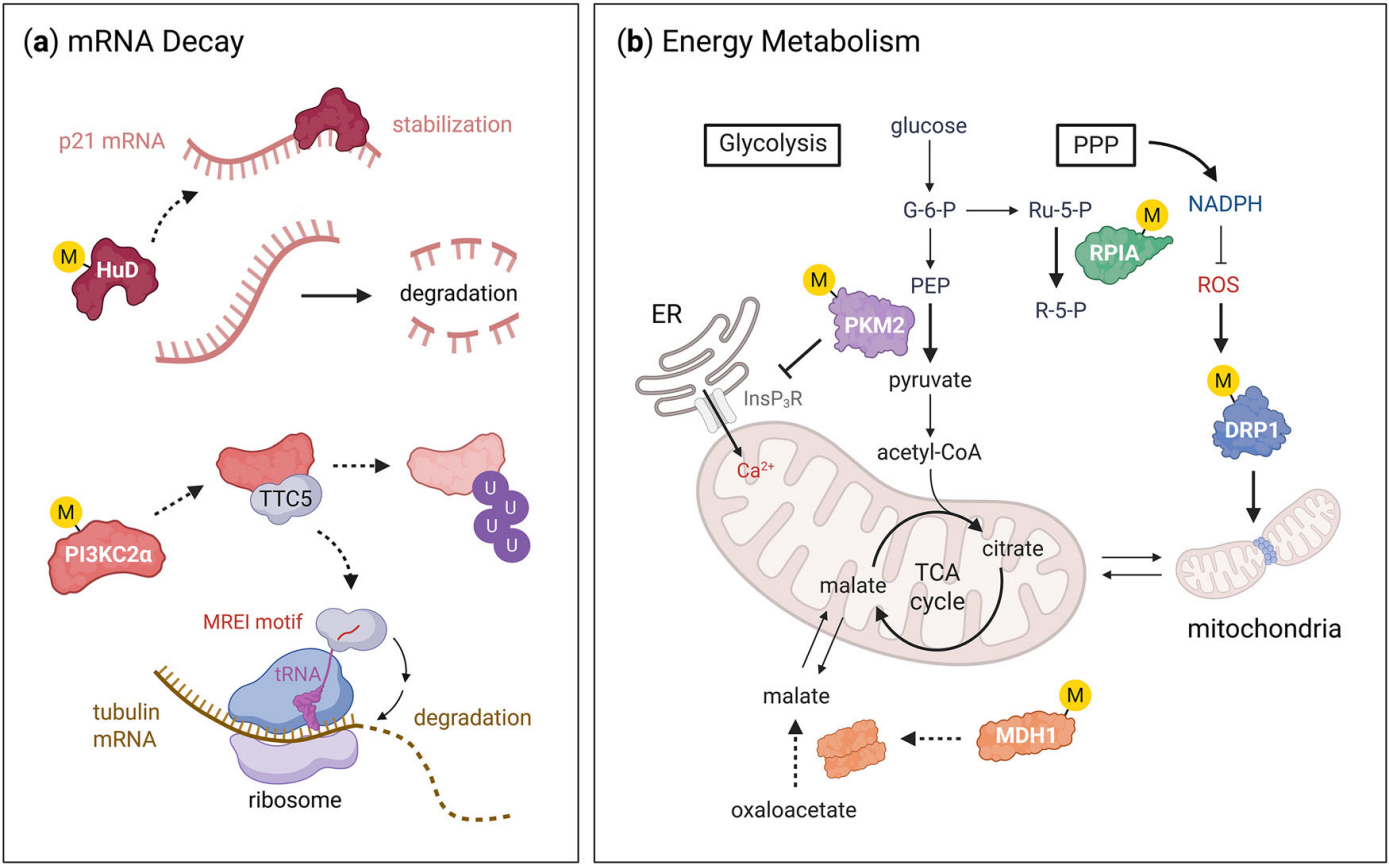
Cytoplasmic functions of CARM1. **a** Regulation of mRNA decay by CARM1. CARM1 promotes mRNA degradation by methylating HuD, which leads to its dissociation from the 3′ untranslated region of *CDKN1A* (p21) mRNA, thus destabilizing the transcript. In addition, CARM1 methylates PI3KC2α, promoting its degradation through the ubiquitin–proteasome system. This degradation event subsequently initiates TTC5-mediated tubulin autoregulation through recognition of the nascent N-terminal MREI motif. **b** Regulation of energy metabolism by CARM1. CARM1 orchestrates metabolic reprogramming through arginine methylation of key metabolic enzymes. CARM1 methylates PKM2, resulting in reduced oxidative phosphorylation and a shift toward aerobic glycolysis. It also inhibits glutamine metabolism by methylating and suppressing MDH1 activity. In the pentose phosphate pathway (PPP), CARM1 methylates RPIA, altering NADPH production and redox balance. This contributes to the adaptive redox homeostasis of tumor cells under oxidative stress. Furthermore, CARM1 methylates DRP1, thereby promoting mitochondrial fission. CARM1 also indirectly affects mitochondrial calcium uptake through regulation of InsP₃R-mediated Ca²⁺ release from the ER. G-6-P, glucose 6-phosphate; NADPH, nicotinamide adenine dinucleotide phosphate (reduced); PEP, phosphoenolpyruvate; R-5-P, ribose 5-phosphate; Ru-5-P, ribulose-5-phosphate.

In addition to its well-characterized nuclear functions, CARM1 is also localized in the cytoplasm, where it regulates diverse cellular processes through the methylation of nonhistone substrates, including HuD^99^, PI3KC2α^29^, PKM2^25,26^, MDH1^27^, ribose 5-phosphate isomerase (RPIA)^100^ and DRP1^4^. For example, CARM1 knockdown stabilizes *CDKN1A* mRNA by enhancing HuD binding to its 3′ untranslated region)^99^. In parallel, CARM1 methylates PI3KC2α and promotes its degradation through proteasome-ubiquitination mechanism, thereby initiating TTC5-mediated tubulin autoregulation^29^ (Fig. 5a). These findings indicate that in addition to nuclear CARM1 promoting mRNA degradation through the nonsense-mediated decay pathway^92^, cytoplasmic CARM1 also regulates mRNA stability. CARM1 also modulates cellular metabolism by methylating key enzymes. PKM2 methylation enhances its enzymatic activity^26^ and inhibits inositol-1,4,5-trisphosphate receptor (InsP₃R)-mediated calcium influx from the endoplasmic reticulum (ER) to the mitochondria^25^, thereby shifting energy metabolism toward aerobic glycolysis (that is, the Warburg effect). Similarly, the methylation of MDH1 favors its monomeric, enzymatically inactive form, resulting in impaired mitochondrial respiration, glutamine metabolism and nicotinamide adenine dinucleotide phosphate (NADPH) generation, ultimately disrupting redox homeostasis^27^. Notably, oxidative stress dynamically alters the substrate specificity of CARM1. Under redox stress, MDH1 methylation is reduced^27^, whereas RPIA and DRP1 methylation increases^4,39,100^. Methylated RPIA enhances NADPH production through the pentose phosphate pathway, promoting ROS detoxification and supporting cell survival^100^. In contrast, DRP1 methylation facilitates mitochondrial fission and increases ROS production, thereby driving cellular senescence^4^ (Fig. 5b). Collectively, these observations establish CARM1 as a redox-sensitive regulator that orchestrates opposing cellular outcomes, such as cell survival versus senescence, through the selective methylation of distinct substrates under oxidative stress.

## Nonenzymatic, scaffolding functions

Several enzymes exhibit scaffolding functions in addition to their catalytic activity^101^, and CARM1 is no exception. Although early studies comparing enzyme-dead CARM1 KI mice with full CARM1 KO models showed that the physiological roles of CARM1 are mediated by its methyltransferase activity^31^, accumulating evidence now supports a dual-function model wherein CARM1 also exerts essential noncatalytic scaffolding roles that contribute to cellular homeostasis^32–35^.

**Fig. 6:**
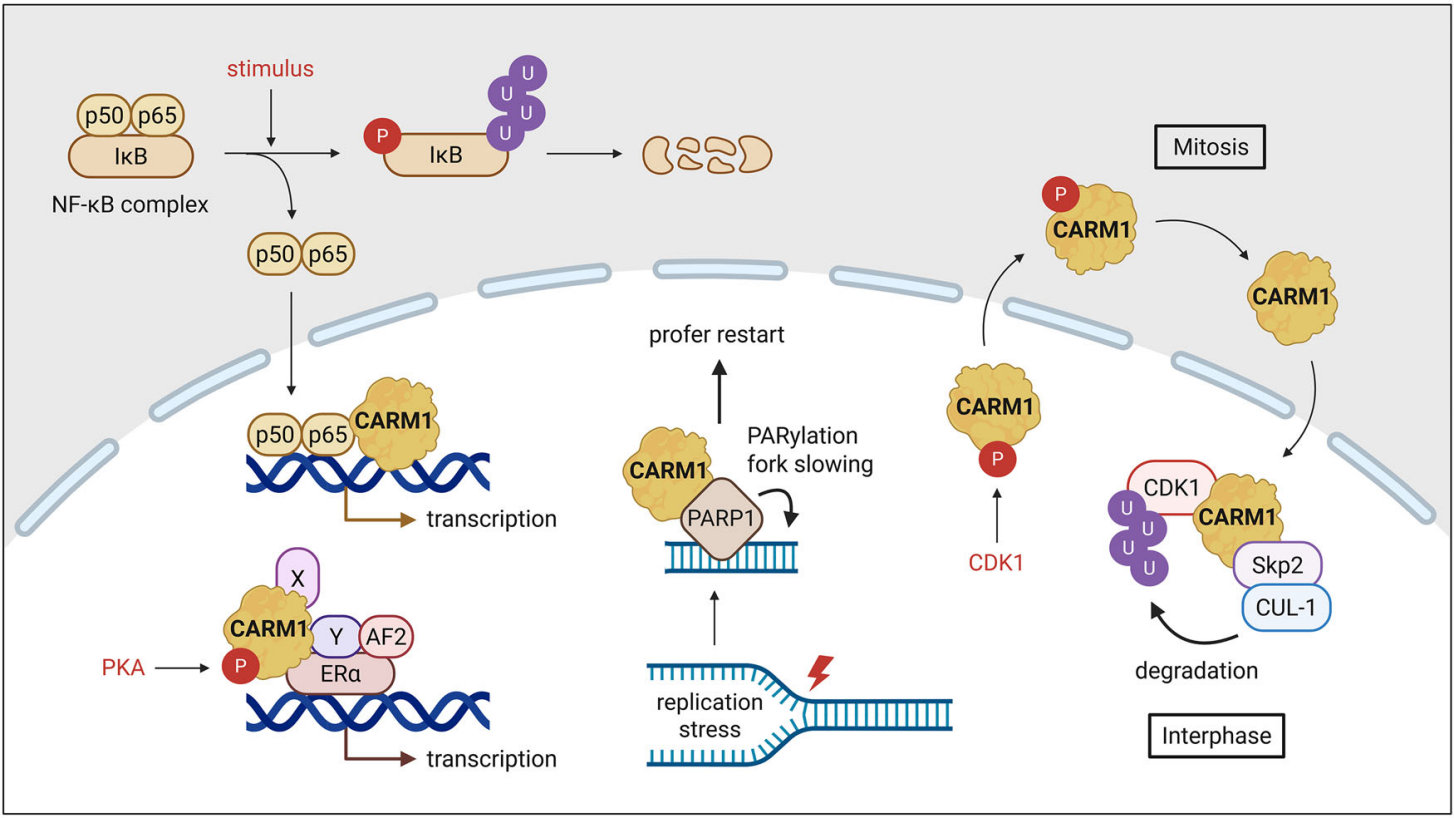
Scaffolding functions of CARM1. CARM1 functions as a scaffold protein in a context-dependent manner. In the NF-κB signaling pathway, CARM1 promotes the expression of NF-κB target genes in response to TNF or PMA/ionomycin stimulation, independently of its methyltransferase activity. In estrogen signaling, CARM1 acts in a methylation-dependent fashion, interacting with ERα. By contrast, in the cAMP signaling pathway, phosphorylation of CARM1 at Ser447 by PKA enables it to function as a pioneer factor in a methylation-independent manner. Under replication stress, CARM1 facilitates PARP1-mediated PARylation at replication forks, promoting fork reversal and replication restart. In addition, during interphase, CARM1 serves as a scaffold that bridges CDK1 to the Skp2/CUL-1 E3 ubiquitin ligase complex, thereby facilitating CDK1 ubiquitination and proteasomal degradation.

Initial observations showed that CARM1 regulates the expression of several NF-κB target genes in response to TNF and PMA/ionomycin stimulation independently of its enzymatic activity. Although CARM1 does not affect the recruitment of RelA/p65 to chromatin, it facilitates transcriptional activation by stabilizing the pre-initiation complex, functioning as a molecular scaffold that promotes protein–protein interactions at target gene promoters^32^. Further mechanistic insight into the noncatalytic roles of CARM1 emerged from studies investigating ER-mediated transcription. Although its catalytic activity and the resulting H3R17 methylation are essential for the assembly of the ERα–coactivator complex in response to estrogen, CARM1 is dispensable for transcriptional responses to cAMP signaling. In the latter context, PKA-mediated phosphorylation at S447 enables CARM1 to interact with unliganded ERα and function as a pioneer factor that primes chromatin for transcriptional activation, independent of its methyltransferase activity^33^. More recently, nonenzymatic functions of CARM1 have been implicated in DNA replication and cell cycle control. CARM1 interacts with PARP1 and promotes PARylation at the replication forks in a methylation-independent manner, facilitating fork slowing and reversal in response to replication stress^34^. In addition, CARM1 serves as a nuclear adapter that bridges CDK1 to the Skp2/CUL-1 E3 ubiquitin ligase complex, promoting CDK1 ubiquitination and degradation. Given the pivotal role of CDK1 in G2/M transition, this scaffolding function has important implications for cell cycle progression and cellular proliferation^35^ (Fig. 6).

Collectively, these findings underscore the multifaceted nature of CARM1 as both a methyltransferase and structural coordinator of signaling networks. This dual functionality has important implications for therapeutic strategies. To fully modulate CARM1 activity in disease contexts, it may be necessary to develop highly selective enzymatic inhibitors as well as design approaches that directly degrade or sequester the protein, thereby abolishing both its catalytic and scaffolding functions.

## Current status of CARM1 inhibitor development

**Fig. 7:**
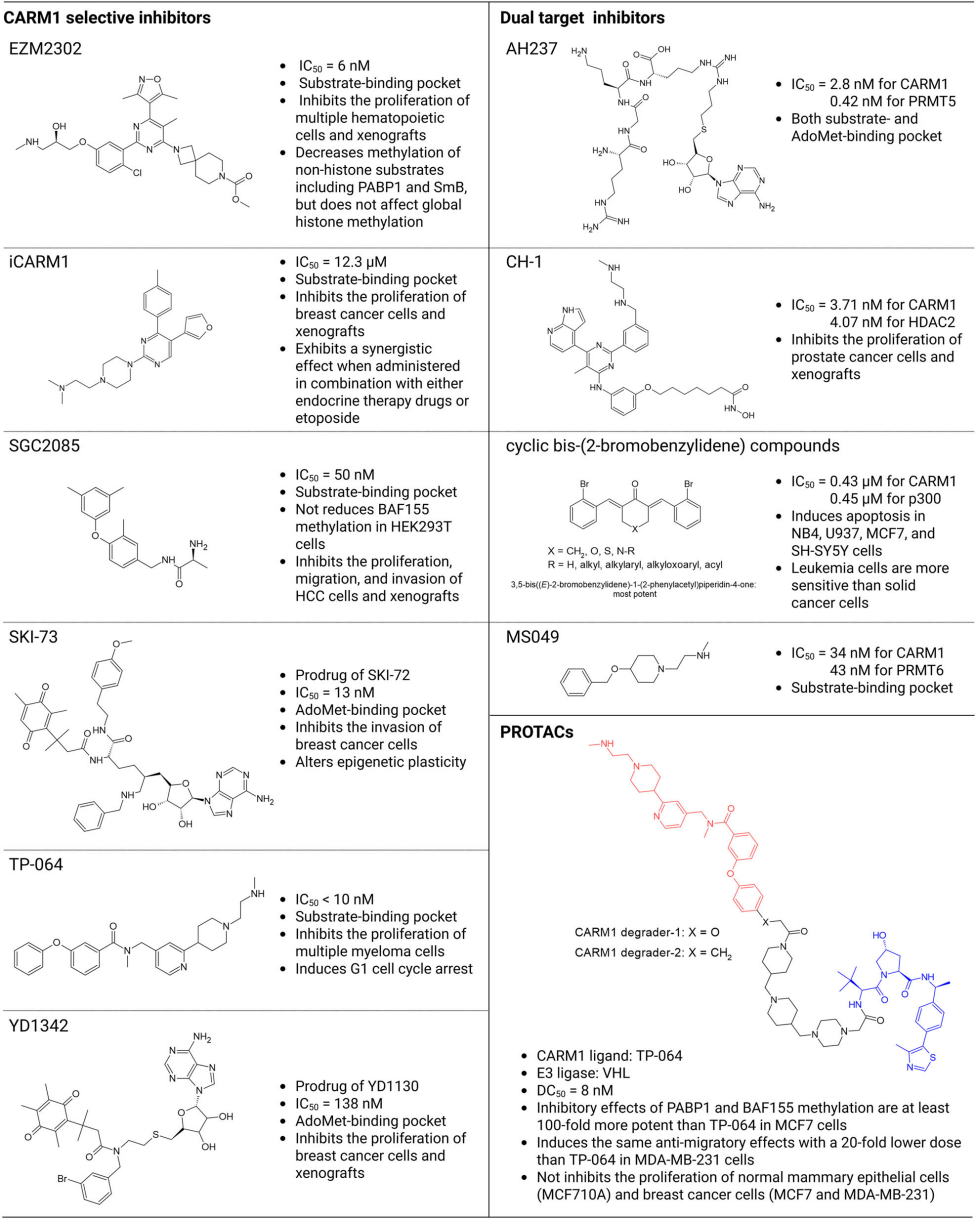
Currently developed CARM1 inhibitors and PROTACs. Summary of the chemical structures and key features of selective, dual-target and PROTAC-based CARM1 inhibitors. DC_50_, half-maximal degradation concentration; IC_50_, half-maximum inhibitory concentration.

As described above, aberrant expression or activity of CARM1 has been implicated in the initiation and progression of various cancers^36,37^, making it an attractive target for anticancer therapy. Therefore, considerable efforts have been directed toward the development of selective CARM1 inhibitors. However, clinical translation remains limited owing to modest in vivo efficacy and the inability of current inhibitors to target the nonenzymatic functions of CARM1. To overcome these challenges, strategies have recently shifted toward the development of dual-function inhibitors and PROTACs. A summary of representative CARM1 inhibitors and degraders is presented in Fig. 7.

## Chemical inhibitors

Many small-molecule CARM1 inhibitors have been developed since 2016, including SGC2085^102^, EZM2302^43^, TP-064^47^, SKI-73^103^, iCARM1^104^ and YD1342^105^, several of which have exhibited antitumor activity in preclinical models. SGC2085 inhibited the proliferation and invasion of hepatocellular carcinoma cells in vitro and suppressed tumor growth in vivo^102^. EZM2302 and TP-064 revealed antiproliferative effects in hematologic malignancies, with EZM2302 showing pronounced efficacy in multiple myeloma models, both in vitro and in xenografts^43,47^. In breast cancer models, SKI-73 suppressed cell invasion without affecting cell proliferation^103^, whereas iCARM1 and YD1342 exhibited robust growth inhibitory effects in vitro and in vivo^104,105^. These functional differences probably stem from their distinct binding mechanisms and chemical structures despite targeting the enzymatic activity of CARM1.

Most CARM1 inhibitors compete with either AdoMet or its substrates. SKI-73 and YD1342 (prodrugs of SKI-72 and YD1130, respectively) target the highly conserved AdoMet-binding pocket, limiting their selectivity^103,105^. In contrast, SGC2085, EZM2302, TP-064 and iCARM1 bind to the substrate-binding site, providing greater selectivity across the PRMT family^43,47,102,104^. Among these, iCARM1, a structure-guided inhibitor developed based on the PH6 pharmacophore model, exhibited improved specificity and activity compared with earlier agents such as EZM2302 and TP-064. Its rational design incorporates key interactions including π–π stacking, salt bridges and hydrogen bonding within the substrate-binding cleft^104^. Nevertheless, all the reported CARM1 inhibitors remain in the preclinical stage as chemical probes, underscoring the need for continued optimization for clinical development. Given the divergent cellular effects of structurally distinct CARM1 inhibitors—such as TP-064’s inhibition of nuclear histone methylation versus EZM2302’s selectivity for cytoplasmic substrates—future therapeutic strategies should go beyond enzymatic potency alone. Instead, emphasis should be placed on the substrate specificity and subcellular targeting. Mechanistically guided selection of inhibitors is essential for tailoring treatment to the disease context, thereby enhancing therapeutic benefits, while reducing off-target effects.

## Multitarget inhibitors

Given the functional redundancy and cooperation among epigenetic regulators, multitarget approaches have emerged as promising therapeutic strategies. In 2020, a series of cyclic bis(2-bromobenzylidene) analogs were developed as dual inhibitors of CARM1 and CBP/p300, inducing apoptosis in various cancer cell lines, including MCF7, NB4, U937 and SH-SY5Y^106^. These findings are consistent with previous reports showing the synergistic effects of CBP/p300 and CARM1 co-inhibition in diffuse large B cell lymphoma^107^. More recently, CH-1 was developed in 2023 as a dual inhibitor targeting CARM1 and HDAC2, revealing potent antiproliferative activity in prostate cancer models both in vitro and in vivo^108^. In parallel, pan-PRMT inhibitors, such as MS049^109^ and AH237^110^, have been developed to co-target CARM1 and other members of the PRMT family. MS049, a dual inhibitor of CARM1 and PRMT6, reduced H3R2me2a and H3R17me2a. Although its antitumor activity remains under investigation, dual inhibition of mitosis-regulating PRMTs may confer synergistic therapeutic benefits^109^. AH237 exhibits broad inhibitory activity against CARM1 and PRMT5, and although not yet evaluated in cancer models, its ability to reduce ADMA and SDMA levels suggests potential utility^110^, particularly in combination with agents such as GSK3368715 (PRMT1 inhibitor) and GSK3326595 (PRMT5 inhibitor), which show synergistic antitumor effects^111^. Collectively, these studies indicate that CARM1-based multitarget inhibition, particularly in combination with other epigenetic regulators, may be a more effective therapeutic strategy than single-agent approaches. However, further validation using diverse in vivo cancer models is required to assess the translational potential.

## PROTACs

Although conventional small-molecule inhibitors of CARM1 function by blocking its enzymatic activity, emerging evidence of the noncatalytic roles of CARM1 in transcriptional scaffolding, protein–protein interactions and chromatin architecture necessitates approaches that eliminate the protein. Therefore, CARM1-targeting PROTACs have recently been developed. Two PROTAC molecules, CARM1 degrader-1 and CARM1 degrader-2, were identified in 2023^48^. Both use TP-064 as the CARM1-binding ligand linked to a Von Hippel–Lindau E3 ligase ligand. These PROTACs induced the rapid and efficient degradation of CARM1 within several hours of treatment. Notably, CARM1 degrader-1 exhibited over 100-fold increased potency in suppressing CARM1-mediated methylation relative to TP-064, and achieved comparable inhibition of cancer cell migration at 20-fold lower concentrations in breast cancer cells^48^. These findings highlight the therapeutic potential of PROTAC-based strategies that can simultaneously abrogate the enzymatic and nonenzymatic functions of CARM1. Further development and optimization of CARM1 PROTACs may represent a transformative approach for targeting this multifaceted oncogenic regulator.

## Future perspectives and conclusions

Beyond its well-established nuclear role as a histone methyltransferase that regulates transcription and chromatin dynamics, accumulating evidence has revealed that CARM1 also performs critical nonhistone and nonnuclear functions. These include the regulation of RNA metabolism, modulation of microtubule dynamics and maintenance of cellular homeostasis through methylation of nonhistone proteins involved in energy metabolism and cytoskeletal organization. Additionally, CARM1 functions as a scaffolding protein independent of its enzymatic activity, facilitating the assembly of multiprotein complexes that are essential for intracellular signaling and structural integrity. These emerging roles underscore the functional complexity of CARM1 and highlight the limitations of conventional catalytic inhibitors, which fail to target nonenzymatic and compartment-specific functions. CARM1 is frequently overexpressed in certain cancers and exhibits stimulus-dependent subcellular relocalization, potentially contributing to pathological alterations. These observations indicate that its enzymatic hyperactivity as well as the aberrant expression and mislocalization of CARM1 play crucial roles in disease pathogenesis. Although numerous selective CARM1 inhibitors have been developed and evaluated in preclinical settings, none has progressed to clinical trials, implying that enzymatic inhibition alone may be insufficient for therapeutic efficacy. Furthermore, the indiscriminate suppression of the physiological functions of CARM1 may result in unintended adverse effects.

To overcome these limitations, targeted protein degradation technologies such as PROTACs have emerged as promising alternatives. Unlike catalytic inhibitors, PROTACs induce the degradation of the entire CARM1 protein, thereby abolishing both enzymatic and scaffolding functions. For instance, a recently reported CARM1 degrader has the capacity to disrupt CARM1-dependent protein complexes. Although its full pharmacological profile remains to be elucidated, the CARM1 degrader serves as a proof-of-concept for the comprehensive functional targeting of CARM1. One notable research direction is the development of organelle-specific CARM1 degraders. By selectively targeting CARM1 in the cytoplasm or other compartments, these strategies can dissect compartmentalized functions, while sparing essential nuclear activities. This approach may expand the therapeutic window, particularly in nononcological diseases, such as age-related disorders or metabolic syndromes, where nonnuclear CARM1 functions appear to be physiologically relevant.

Furthermore, rational combination therapies that co-target CARM1 along with other epigenetic or post-translational regulators, such as CBP/p300, PRMT5 and HDACs, may uncover synthetic vulnerabilities and improve therapeutic specificity. To fully harness the potential of these approaches, a deeper understanding of the dynamic interactome, subcellular distribution, and post-translational regulation of CARM1 is essential. Advances in proteomics, spatial omics and high-resolution imaging are critical for delineating the context-specific CARM1 functions across cellular compartments and disease states. Concurrently, identifying predictive biomarkers of CARM1 dependence, particularly those associated with its noncatalytic role, will be instrumental in patient stratification in future clinical trials.

In summary, the expanding landscape of CARM1 biology requires a paradigm shift from conventional enzymatic inhibition to comprehensive function-oriented degradation strategies. The integration of protein degradation technologies, organelle-specific targeting and systems-level analysis provides a transformative path toward next-generation CARM1-targeted therapies for cancer and beyond.

## References

[1] Paik, W. K. & Kim, S. Enzymatic methylation of protein fractions from calf thymus nuclei. *Biochem. Biophys. Res. Commun.* **29**, 14–20 (1967). 6055181 10.1016/0006-291x(67)90533-5

[2] Kim, S. et al. PRMT6-mediated H3R2me2a guides Aurora B to chromosome arms for proper chromosome segregation. *Nat. Commun.* **11**, 612 (2020). 32001712 10.1038/s41467-020-14511-w PMC6992762

[3] Hwang, J. W. et al. PRMT5 promotes DNA repair through methylation of 53BP1 and is regulated by Src-mediated phosphorylation. *Commun. Biol.* **3**, 428 (2020). 32759981 10.1038/s42003-020-01157-z PMC7406651

[4] Cho, Y. & Kim, Y. K. ROS-mediated cytoplasmic localization of CARM1 induces mitochondrial fission through DRP1 methylation. *Redox Biol.* **73**, 103212 (2024). 38838552 10.1016/j.redox.2024.103212 PMC11179627

[5] Troffer-Charlier, N., Cura, V., Hassenboehler, P., Moras, D. & Cavarelli, J. Functional insights from structures of coactivator-associated arginine methyltransferase 1 domains. *EMBO J.* **26**, 4391–4401 (2007). 17882262 10.1038/sj.emboj.7601855 PMC2034665

[6] Yue, W. W., Hassler, M., Roe, S. M., Thompson-Vale, V. & Pearl, L. H. Insights into histone code syntax from structural and biochemical studies of CARM1 methyltransferase. *EMBO J.* **26**, 4402–4412 (2007). 17882261 10.1038/sj.emboj.7601856 PMC2034666

[7] Lin, W. J., Gary, J. D., Yang, M. C., Clarke, S. & Herschman, H. R. The mammalian immediate-early TIS21 protein and the leukemia-associated BTG1 protein interact with a protein–arginine *N*-methyltransferase. *J. Biol. Chem.* **271**, 15034–15044 (1996). 8663146 10.1074/jbc.271.25.15034

[8] Scott, H. S. et al. Identification and characterization of two putative human arginine methyltransferases (HRMT1L1 and HRMT1L2). *Genomics* **48**, 330–340 (1998). 9545638 10.1006/geno.1997.5190

[9] Tang, J., Gary, J. D., Clarke, S. & Herschman, H. R. PRMT 3, a type I protein arginine *N*-methyltransferase that differs from PRMT1 in its oligomerization, subcellular localization, substrate specificity, and regulation. *J. Biol. Chem.* **273**, 16935–16945 (1998). 9642256 10.1074/jbc.273.27.16935

[10] Chen, D. et al. Regulation of transcription by a protein methyltransferase. *Science* **284**, 2174–2177 (1999). 10381882 10.1126/science.284.5423.2174

[11] Pollack, B. P. et al. The human homologue of the yeast proteins Skb1 and Hsl7p interacts with Jak kinases and contains protein methyltransferase activity. *J. Biol. Chem.* **274**, 31531–31542 (1999). 10531356 10.1074/jbc.274.44.31531

[12] Frankel, A. et al. The novel human protein arginine *N*-methyltransferase PRMT6 is a nuclear enzyme displaying unique substrate specificity. *J. Biol. Chem.* **277**, 3537–3543 (2002). 11724789 10.1074/jbc.M108786200

[13] Miranda, T. B., Miranda, M., Frankel, A. & Clarke, S. PRMT7 is a member of the protein arginine methyltransferase family with a distinct substrate specificity. *J. Biol. Chem.* **279**, 22902–22907 (2004). 15044439 10.1074/jbc.M312904200

[14] Lee, J., Sayegh, J., Daniel, J., Clarke, S. & Bedford, M. T. PRMT8, a new membrane-bound tissue-specific member of the protein arginine methyltransferase family. *J. Biol. Chem.* **280**, 32890–32896 (2005). 16051612 10.1074/jbc.M506944200

[15] Lee, H. W., Kim, S. & Paik, W. K. *S*-Adenosylmethionine: protein-arginine methyltransferase. Purification and mechanism of the enzyme. *Biochemistry* **16**, 78–85 (1977). 12796 10.1021/bi00620a013

[16] Hwang, J. W., Cho, Y., Bae, G., Kim, S. & Kim, Y. K. Protein arginine methyltransferases: promising targets for cancer therapy. *Exp. Mol. Med.* **53**, 788–808 (2021). 34006904 10.1038/s12276-021-00613-y PMC8178397

[17] Schurter, B. T. et al. Methylation of histone H3 by coactivator-associated arginine methyltransferase 1. *Biochemistry* **40**, 5747–5756 (2001). 11341840 10.1021/bi002631b

[18] Jacques, S. L. et al. CARM1 preferentially methylates H3R17 over H3R26 through a random kinetic mechanism. *Biochemistry* **55**, 1635–1644 (2016). 26848779 10.1021/acs.biochem.5b01071

[19] Bauer, U., Daujat, S., Nielsen, S. J., Nightingale, K. & Kouzarides, T. Methylation at arginine 17 of histone H3 is linked to gene activation. *EMBO Rep.* **3**, 39–44 (2002). 11751582 10.1093/embo-reports/kvf013 PMC1083932

[20] Lee, J. & Bedford, M. T. PABP1 identified as an arginine methyltransferase substrate using high-density protein arrays. *EMBO Rep.* **3**, 268–273 (2002). 11850402 10.1093/embo-reports/kvf052 PMC1084016

[21] Hu, S. et al. Protein arginine methyltransferase CARM1 attenuates the paraspeckle-mediated nuclear retention of mRNAs containing IRAlus. *Genes Dev.* **29**, 630–645 (2015). 25792598 10.1101/gad.257048.114 PMC4378195

[22] Cheng, D., Cote, J., Shaaban, S. & Bedford, M. T. The arginine methyltransferase CARM1 regulates the coupling of transcription and mRNA processing. *Mol. Cell* **25**, 71–83 (2007). 17218272 10.1016/j.molcel.2006.11.019

[23] Wang, L. et al. MED12 methylation by CARM1 sensitizes human breast cancer cells to chemotherapy drugs. *Sci. Adv.* **1**, e1500463 (2015). 26601288 10.1126/sciadv.1500463 PMC4646802

[24] Feng, Q., Yi, P., Wong, J. & O’Malley, B. W. Signaling within a coactivator complex: methylation of SRC-3/AIB1 is a molecular switch for complex disassembly. *Mol. Cell. Biol.* **26**, 7846–7857 (2006). 16923966 10.1128/MCB.00568-06 PMC1636757

[25] Liu, F. et al. PKM2 methylation by CARM1 activates aerobic glycolysis to promote tumorigenesis. *Nat. Cell Biol.* **19**, 1358–1370 (2017). 29058718 10.1038/ncb3630 PMC5683091

[26] Abeywardana, T. et al. CARM1 suppresses de novo serine synthesis by promoting PKM2 activity. *J. Biol. Chem.* **293**, 15290–15303 (2018). 30131339 10.1074/jbc.RA118.004512 PMC6166735

[27] Wang, Y. et al. Arginine methylation of MDH1 by CARM1 inhibits glutamine metabolism and suppresses pancreatic cancer. *Mol. Cell* **64**, 673–687 (2016). 27840030 10.1016/j.molcel.2016.09.028

[28] Zhong, X. et al. CARM1 Methylates GAPDH to regulate glucose metabolism and is suppressed in liver cancer. *Cell. Rep.* **24**, 3207–3223 (2018). 30232003 10.1016/j.celrep.2018.08.066

[29] Cho, Y. et al. CARM1 regulates tubulin autoregulation through PI3KC2alpha R175 methylation. *Cell. Commun. Signal.* **23**, 120–z (2025). 40045375 10.1186/s12964-025-02124-z PMC11884010

[30] Suresh, S., Huard, S. & Dubois, T. CARM1/PRMT4: making its mark beyond its function as a transcriptional coactivator. *Trends Cell Biol.* **31**, 402–417 (2021). 33485722 10.1016/j.tcb.2020.12.010

[31] Kim, D. et al. Enzymatic activity is required for the in vivo functions of CARM1. *J. Biol. Chem.* **285**, 1147–1152 (2010). 19897492 10.1074/jbc.M109.035865 PMC2801243

[32] Jayne, S., Rothgiesser, K. M. & Hottiger, M. O. CARM1 but not its enzymatic activity is required for transcriptional coactivation of NF-kappaB-dependent gene expression. *J. Mol. Biol.* **394**, 485–495 (2009). 19769987 10.1016/j.jmb.2009.09.032

[33] Carascossa, S., Dudek, P., Cenni, B., Briand, P. & Picard, D. CARM1 mediates the ligand-independent and tamoxifen-resistant activation of the estrogen receptor alpha by cAMP. *Genes Dev.* **24**, 708–719 (2010). 20360387 10.1101/gad.568410 PMC2849127

[34] Genois, M. et al. CARM1 regulates replication fork speed and stress response by stimulating PARP1. *Mol. Cell* **81**, 784–800.e8 (2021). 33412112 10.1016/j.molcel.2020.12.010 PMC7897296

[35] Cho, Y., Song, D., Kim, S. & Kim, Y. K. CARM1 S217 phosphorylation by CDK1 in late G2 phase facilitates mitotic entry. *Cell. Death Dis.* **16**, 202–z (2025). 40133267 10.1038/s41419-025-07533-z PMC11937338

[36] Santos, M., Hwang, J. W. & Bedford, M. T. CARM1 arginine methyltransferase as a therapeutic target for cancer. *J. Biol. Chem.* **299**, 105124 (2023). 37536629 10.1016/j.jbc.2023.105124 PMC10474102

[37] Liu, K. et al. The oncogenic role and immune infiltration for CARM1 identified by pancancer analysis. *J. Oncol.* **2021**, 2986444 (2021). 34745258 10.1155/2021/2986444 PMC8566078

[38] Wang, S. M., Dowhan, D. H., Eriksson, N. A. & Muscat, G. E. O. CARM1/PRMT4 is necessary for the glycogen gene expression programme in skeletal muscle cells. *Biochem. J.* **444**, 323–331 (2012). 22428544 10.1042/BJ20112033

[39] Cho, Y. & Kim, Y. K. CARM1 phosphorylation at S595 by p38gamma MAPK drives ROS-mediated cellular senescence. *Redox Biol.* **76**, 103344 (2024). 39265499 10.1016/j.redox.2024.103344 PMC11415932

[40] Pang, L. et al. Loss of CARM1 is linked to reduced HuR function in replicative senescence. *BMC Mol. Biol.* **14**, 15–15 (2013). 23837869 10.1186/1471-2199-14-15 PMC3718661

[41] Habashy, H. O., Rakha, E. A., Ellis, I. O. & Powe, D. G. The oestrogen receptor coactivator CARM1 has an oncogenic effect and is associated with poor prognosis in breast cancer. *Breast Cancer Res. Treat.* **140**, 307–316 (2013). 23887673 10.1007/s10549-013-2614-y

[42] Cheng, H. et al. Overexpression of CARM1 in breast cancer is correlated with poorly characterized clinicopathologic parameters and molecular subtypes. *Diagn. Pathol.* **8**, 129–129 (2013). 23915145 10.1186/1746-1596-8-129 PMC3766166

[43] Drew, A. E. et al. Identification of a CARM1 inhibitor with potent in vitro and in vivo activity in preclinical models of multiple myeloma. *Sci. Rep.* **7**, 17993–z (2017). 29269946 10.1038/s41598-017-18446-z PMC5740082

[44] Greenblatt, S. M. et al. CARM1 is essential for myeloid leukemogenesis but dispensable for normal hematopoiesis. *Cancer Cell.* **33**, 1111–1127.e5 (2018). 29894694 10.1016/j.ccell.2018.05.007 PMC6191185

[45] Wang, F. et al. Nup54-induced CARM1 nuclear importation promotes gastric cancer cell proliferation and tumorigenesis through transcriptional activation and methylation of Notch2. *Oncogene* **41**, 246–259 (2022). 34725461 10.1038/s41388-021-02078-9

[46] Gao, G. et al. The NFIB/CARM1 partnership is a driver in preclinical models of small cell lung cancer. *Nat. Commun.* **14**, 363–y (2023). 36690626 10.1038/s41467-023-35864-y PMC9870865

[47] Nakayama, K. et al. TP-064, a potent and selective small molecule inhibitor of PRMT4 for multiple myeloma. *Oncotarget* **9**, 18480–18493 (2018). 29719619 10.18632/oncotarget.24883 PMC5915086

[48] Xie, H. et al. Development of potent and selective coactivator-associated arginine methyltransferase 1 (CARM1) degraders. *J. Med. Chem.* **66**, 13028–13042 (2023). 37703322 10.1021/acs.jmedchem.3c00982 PMC10775954

[49] Yadav, N. et al. Specific protein methylation defects and gene expression perturbations in coactivator-associated arginine methyltransferase 1-deficient mice. *Proc. Natl Acad. Sci. USA* **100**, 6464–6468 (2003). 12756295 10.1073/pnas.1232272100 PMC164469

[50] O’Brien, K. B. et al. CARM1 is required for proper control of proliferation and differentiation of pulmonary epithelial cells. *Development* **137**, 2147–2156 (2010). 20530543 10.1242/dev.037150 PMC2882134

[51] Kim, J. et al. Loss of CARM1 results in hypomethylation of thymocyte cyclic AMP-regulated phosphoprotein and deregulated early T cell development. *J. Biol. Chem.* **279**, 25339–25344 (2004). 15096520 10.1074/jbc.M402544200

[52] Yadav, N. et al. CARM1 promotes adipocyte differentiation by coactivating PPARgamma. *EMBO Rep.* **9**, 193–198 (2008). 18188184 10.1038/sj.embor.7401151 PMC2246418

[53] Ito, T. et al. Arginine methyltransferase CARM1/PRMT4 regulates endochondral ossification. *BMC Dev. Biol.* **9**, 47–47 (2009). 19725955 10.1186/1471-213X-9-47 PMC2754437

[54] Kawabe, Y., Wang, Y. X., McKinnell, I. W., Bedford, M. T. & Rudnicki, M. A. Carm1 regulates Pax7 transcriptional activity through MLL1/2 recruitment during asymmetric satellite stem cell divisions. *Cell Stem Cell* **11**, 333–345 (2012). 22863532 10.1016/j.stem.2012.07.001 PMC3438319

[55] Stouth, D. W. et al. CARM1 regulates AMPK signaling in skeletal muscle. *iScience* **23**, 101755 (2020). 33241200 10.1016/j.isci.2020.101755 PMC7672286

[56] Bao, J. et al. The arginine methyltransferase CARM1 represses p300*ACT*CREMtau activity and is required for spermiogenesis. *Nucleic Acids Res.* **46**, 4327–4343 (2018). 29659998 10.1093/nar/gky240 PMC5961101

[57] Bao, J. et al. Mouse models of overexpression reveal distinct oncogenic roles for different type I protein arginine methyltransferases. *Cancer Res.* **79**, 21–32 (2019). 30352814 10.1158/0008-5472.CAN-18-1995 PMC6714580

[58] Thandapani, P., O’Connor, T. R., Bailey, T. L. & Richard, S. Defining the RGG/RG motif. *Mol. Cell* **50**, 613–623 (2013). 23746349 10.1016/j.molcel.2013.05.021

[59] Shishkova, E. et al. Global mapping of CARM1 substrates defines enzyme specificity and substrate recognition. *Nat. Commun.* **8**, 15571 (2017). 28537268 10.1038/ncomms15571 PMC5458078

[60] Gao, W. et al. JMJD6 licenses ERα-dependent enhancer and coding gene activation by modulating the recruitment of the CARM1/MED12 co-activator complex. *Mol. Cell* **70**, 340–357.e8 (2018). 29628309 10.1016/j.molcel.2018.03.006 PMC6258263

[61] Wang, L. et al. CARM1 automethylation is controlled at the level of alternative splicing. *Nucleic Acids Res*. **41**, 6870–6880 (2013). 23723242 10.1093/nar/gkt415 PMC3737532

[62] Shlensky, D. et al. Differential CARM1 isoform expression in subcellular compartments and among malignant and benign breast tumors. *PLoS ONE* **10**, e0128143 (2015). 26030442 10.1371/journal.pone.0128143 PMC4451767

[63] Zheng, M. et al. ESRP1 regulates alternative splicing of CARM1 to sensitize small cell lung cancer cells to chemotherapy by inhibiting TGF-β/Smad signaling. *Aging* **13**, 3554–3572 (2021). 33495408 10.18632/aging.202295 PMC7906186

[64] Peng, B. et al. A hypermethylation strategy utilized by enhancer-bound CARM1 to promote estrogen receptor α-dependent transcriptional activation and breast carcinogenesis. *Theranostics* **10**, 3451–3473 (2020). 32206101 10.7150/thno.39241 PMC7069091

[65] Feng, D. et al. CARM1 drives triple-negative breast cancer progression by coordinating with HIF1A. *Protein Cell* **15**, 744–765 (2024). 38476024 10.1093/procel/pwae010 PMC11443453

[66] Nakayama, N. et al. Cancer-related transcription regulator protein NAC1 forms a protein complex with CARM1 for ovarian cancer progression. *Oncotarget* **9**, 28408–28420 (2018). 29983869 10.18632/oncotarget.25400 PMC6033357

[67] Karakashev, S. et al. CARM1-expressing ovarian cancer depends on the histone methyltransferase EZH2 activity. *Nat. Commun.* **9**, 631 (2018). 29434212 10.1038/s41467-018-03031-3 PMC5809368

[68] Hong, H. et al. Aberrant expression of CARM1, a transcriptional coactivator of androgen receptor, in the development of prostate carcinoma and androgen-independent status. *Cancer* **101**, 83–89 (2004). 15221992 10.1002/cncr.20327

[69] Leonard, S. et al. Arginine methyltransferases are regulated by Epstein–Barr virus in B cells and are differentially expressed in Hodgkin’s lymphoma. *Pathogens* **1**, 52–64 (2012). 25436604 10.3390/pathogens1010052 PMC4235682

[70] Vu, L. P. et al. PRMT4 blocks myeloid differentiation by assembling a methyl-RUNX1-dependent repressor complex. *Cell. Rep.* **5**, 1625–1638 (2013). 24332853 10.1016/j.celrep.2013.11.025 PMC4073674

[71] Zhang, M. et al. Coactivator-associated arginine methyltransferase 1 promotes cell growth and is targeted by microRNA-195-5p in human colorectal cancer. *Tumour Biol.* **39**, 1010428317694305 (2017). 28345460 10.1177/1010428317694305

[72] Ohkura, N., Takahashi, M., Yaguchi, H., Nagamura, Y. & Tsukada, T. Coactivator-associated arginine methyltransferase 1, CARM1, affects pre-mRNA splicing in an isoform-specific manner. *J. Biol. Chem.* **280**, 28927–28935 (2005). 15944154 10.1074/jbc.M502173200

[73] Feng, Q. et al. Biochemical control of CARM1 enzymatic activity by phosphorylation. *J. Biol. Chem.* **284**, 36167–36174 (2009). 19843527 10.1074/jbc.M109.065524 PMC2794732

[74] Higashimoto, K., Kuhn, P., Desai, D., Cheng, X. & Xu, W. Phosphorylation-mediated inactivation of coactivator-associated arginine methyltransferase 1. *Proc. Natl Acad. Sci. USA* **104**, 12318–12323 (2007). 17640894 10.1073/pnas.0610792104 PMC1941467

[75] Lim, C. S. & Alkon, D. L. Protein kinase C stimulates HuD-mediated mRNA stability and protein expression of neurotrophic factors and enhances dendritic maturation of hippocampal neurons in culture. *Hippocampus* **22**, 2303–2319 (2012). 22736542 10.1002/hipo.22048

[76] Chang, N. C. et al. The dystrophin glycoprotein complex regulates the epigenetic activation of muscle stem cell commitment. *Cell Stem Cell* **22**, 755–768.e6 (2018). 29681515 10.1016/j.stem.2018.03.022 PMC5935555

[77] Itonaga, H. et al. Tyrosine phosphorylation of CARM1 promotes its enzymatic activity and alters its target specificity. *Nat. Commun.* **15**, 3415 (2024). 38649367 10.1038/s41467-024-47689-4 PMC11035800

[78] Charoensuksai, P., Kuhn, P., Wang, L., Sherer, N. & Xu, W. O-GlcNAcylation of co-activator-associated arginine methyltransferase 1 regulates its protein substrate specificity. *Biochem. J.* **466**, 587–599 (2015). 25585345 10.1042/BJ20141072

[79] Li, X., Lai, Y., Li, J., Zou, M. & Zou, C. Oxidative stress destabilizes protein arginine methyltransferase 4 via glycogen synthase kinase 3beta to impede lung epithelial cell migration. *Am. J. Physiol. Cell. Physiol.* **313**, C285–C294 (2017). 28637674 10.1152/ajpcell.00073.2017 PMC5625095

[80] Shin, H. R. et al. AMPK–SKP2–CARM1 signalling cascade in transcriptional regulation of autophagy. *Nature* **534**, 553–557 (2016). 27309807 10.1038/nature18014 PMC5568428

[81] Li, C. et al. Nuclear AMPK regulated CARM1 stabilization impacts autophagy in aged heart. *Biochem. Biophys. Res. Commun.* **486**, 398–405 (2017). 28315332 10.1016/j.bbrc.2017.03.053

[82] Kuhn, P. et al. Automethylation of CARM1 allows coupling of transcription and mRNA splicing. *Nucleic Acids Res* **39**, 2717–2726 (2011). 21138967 10.1093/nar/gkq1246 PMC3074151

[83] Chevillard-Briet, M., Trouche, D. & Vandel, L. Control of CBP co-activating activity by arginine methylation. *EMBO J.* **21**, 5457–5466 (2002). 12374746 10.1093/emboj/cdf548 PMC129080

[84] Feng, S. et al. Inhibition of CARM1-mediated methylation of ACSL4 promotes ferroptosis in colorectal cancer. *Adv. Sci.* **10**, e2303484 (2023). 10.1002/advs.202303484 PMC10754121 37946697

[85] Wu, J., Cui, N., Wang, R., Li, J. & Wong, J. A role for CARM1-mediated histone H3 arginine methylation in protecting histone acetylation by releasing corepressors from chromatin. *PLoS ONE* **7**, e34692 (2012). 22723830 10.1371/journal.pone.0034692 PMC3377634

[86] Frietze, S., Lupien, M., Silver, P. A. & Brown, M. CARM1 regulates estrogen-stimulated breast cancer growth through up-regulation of E2F1. *Cancer Res* **68**, 301–306 (2008). 18172323 10.1158/0008-5472.CAN-07-1983

[87] Lee, J. et al. ESRP1-regulated isoform switching of LRRFIP2 determines metastasis of gastric cancer. *Nat. Commun.* **13**, 6274 (2022). 36307405 10.1038/s41467-022-33786-9 PMC9616898

[88] Yu, Y. S. et al. Pontin arginine methylation by CARM1 is crucial for epigenetic regulation of autophagy. *Nat. Commun.* **11**, 6297 (2020). 33293536 10.1038/s41467-020-20080-9 PMC7722926

[89] Wang, L. et al. CARM1 methylates chromatin remodeling factor BAF155 to enhance tumor progression and metastasis. *Cancer Cell.* **25**, 21–36 (2014). 24434208 10.1016/j.ccr.2013.12.007 PMC4004525

[90] Lee, Y., Bedford, M. T. & Stallcup, M. R. Regulated recruitment of tumor suppressor BRCA1 to the p21 gene by coactivator methylation. *Genes Dev.* **25**, 176–188 (2011). 21245169 10.1101/gad.1975811 PMC3022263

[91] Zhao, Z. et al. CARM1-mediated methylation of ASXL2 impairs tumor-suppressive function of MLL3/COMPASS. *Sci. Adv.* **8**, eadd3339 (2022). 36197977 10.1126/sciadv.add3339 PMC9534506

[92] Sanchez, G. et al. A novel role for CARM1 in promoting nonsense-mediated mRNA decay: potential implications for spinal muscular atrophy. *Nucleic Acids Res.* **44**, 2661–2676 (2016). 26656492 10.1093/nar/gkv1334 PMC4824080

[93] Larsen, S. C. et al. Proteome-wide analysis of arginine monomethylation reveals widespread occurrence in human cells. *Sci. Signal.* **9**, rs9 (2016). 27577262 10.1126/scisignal.aaf7329

[94] Zheng, L., Chen, J., Zhou, Z. & He, Z. miR-195 enhances the radiosensitivity of colorectal cancer cells by suppressing CARM1. *Onco Targets Ther.* **10**, 1027–1038 (2017). 28255246 10.2147/OTT.S125067 PMC5325097

[95] Wang, D. & Hu, Y. Long non-coding RNA PVT1 competitively binds microRNA-424-5p to regulate CARM1 in radiosensitivity of non-small-cell lung cancer. *Mol. Ther. Nucleic Acids* **16**, 130–140 (2019). 30861415 10.1016/j.omtn.2018.12.006 PMC6411630

[96] Qin, H., Xu, J., Gong, L., Jiang, B. & Zhao, W. The long noncoding RNA ST7-AS1 promotes laryngeal squamous cell carcinoma by stabilizing CARM1. *Biochem. Biophys. Res. Commun.* **512**, 34–40 (2019). 30853182 10.1016/j.bbrc.2019.02.057

[97] Hupalowska, A. et al. CARM1 and paraspeckles regulate pre-implantation mouse embryo development. *Cell* **175**, 1902–1916.e13 (2018). 30550788 10.1016/j.cell.2018.11.027 PMC6292842

[98] Cheng, D. et al. CARM1 methylates MED12 to regulate its RNA-binding ability. *Life Sci. Alliance* **1**, e201800117 (2018). 30456381 10.26508/lsa.201800117 PMC6238599

[99] Fujiwara, T. et al. CARM1 regulates proliferation of PC12 cells by methylating HuD. *Mol. Cell. Biol.* **26**, 2273–2285 (2006). 16508003 10.1128/MCB.26.6.2273-2285.2006 PMC1430293

[100] Guo, J. et al. Arginine methylation of ribose-5-phosphate isomerase A senses glucose to promote human colorectal cancer cell survival. *Sci. China Life. Sci.* **63**, 1394–1405 (2020). 32157557 10.1007/s11427-019-1562-y

[101] Kung, J. E. & Jura, N. Structural basis for the non-catalytic functions of protein kinases. *Structure* **24**, 7–24 (2016). 26745528 10.1016/j.str.2015.10.020 PMC4706642

[102] Ferreira de Freitas, R. et al. Discovery of a potent and selective coactivator associated arginine methyltransferase 1 (CARM1) inhibitor by virtual screening. *J. Med. Chem.* **59**, 6838–6847 (2016). 27390919 10.1021/acs.jmedchem.6b00668

[103] Cai, X., et al. A chemical probe of CARM1 alters epigenetic plasticity against breast cancer cell invasion. *eLife*10.7554/eLife.47110 (2019). 10.7554/eLife.47110 PMC6917500 31657716

[104] Peng, B. et al. A CARM1 inhibitor potently suppresses breast cancer both in vitro and in vivo. *J. Med. Chem.* **67**, 7921–7934 (2024). 38713486 10.1021/acs.jmedchem.3c02315 PMC11129188

[105] Deng, Y. et al. An adenosine analogue library reveals insights into active sites of protein arginine methyltransferases and enables the discovery of a selective PRMT4 inhibitor. *J. Med. Chem.* **67**, 18053–18069 (2024). 39361813 10.1021/acs.jmedchem.4c01041

[106] Fioravanti, R. et al. Properly substituted cyclic bis-(2-bromobenzylidene) compounds behaved as dual p300/CARM1 inhibitors and induced apoptosis in cancer cells. *Molecules* **25**, 3122 (2020). 32650558 10.3390/molecules25143122 PMC7397249

[107] Veazey, K. J. et al. CARM1 inhibition reduces histone acetyltransferase activity causing synthetic lethality in CREBBP/EP300-mutated lymphomas. *Leukemia* **34**, 3269–3285 (2020). 32576962 10.1038/s41375-020-0908-8 PMC7688486

[108] Liang, S. et al. Discovery and biological evaluation of novel CARM1/HDAC2 dual-targeting inhibitors with anti-prostate cancer agents. *J. Enzym. Inhib. Med. Chem.* **38**, 2241118 (2023). 10.1080/14756366.2023.2241118 PMC10399481 37528657

[109] Shen, Y. et al. Discovery of a potent, selective, and cell-active dual inhibitor of protein arginine methyltransferase 4 and protein arginine methyltransferase 6. *J. Med. Chem.* **59**, 9124–9139 (2016). 27584694 10.1021/acs.jmedchem.6b01033 PMC5063716

[110] Al-Hamashi, A. A., Chen, D., Deng, Y., Dong, G. & Huang, R. Discovery of a potent and dual-selective bisubstrate inhibitor for protein arginine methyltransferase 4/5. *Acta Pharm. Sin. B* **11**, 2709–2718 (2020). 34589391 10.1016/j.apsb.2020.10.013 PMC8463262

[111] Fedoriw, A. et al. Anti-tumor activity of the type I PRMT inhibitor, GSK3368715, synergizes with PRMT5 inhibition through MTAP loss. *Cancer Cell* **36**, 100–114.e25 (2019). 31257072 10.1016/j.ccell.2019.05.014

[112] Xu, W. et al. A transcriptional switch mediated by cofactor methylation. *Science* **294**, 2507–2511 (2001). 11701890 10.1126/science.1065961

[113] Ceschin, D. G. et al. Methylation specifies distinct estrogen-induced binding site repertoires of CBP to chromatin. *Genes Dev.* **25**, 1132–1146 (2011). 21632823 10.1101/gad.619211 PMC3110952

[114] Lee, Y., Coonrod, S. A., Kraus, W. L., Jelinek, M. A. & Stallcup, M. R. Regulation of coactivator complex assembly and function by protein arginine methylation and demethylimination. *Proc. Natl Acad. Sci. USA* **102**, 3611–3616 (2005). 15731352 10.1073/pnas.0407159102 PMC553305

[115] Casadio, F. et al. H3R42me2a is a histone modification with positive transcriptional effects. *Proc. Natl Acad. Sci. USA* **110**, 14894–14899 (2013). 23980157 10.1073/pnas.1312925110 PMC3773778

[116] Goolam, M. et al. Heterogeneity in Oct4 and Sox2 targets biases cell fate in 4-cell mouse embryos. *Cell* **165**, 61–74 (2016). 27015307 10.1016/j.cell.2016.01.047 PMC4819611

[117] Zhang, Z. et al. Crosstalk between histone modifications indicates that inhibition of arginine methyltransferase CARM1 activity reverses HIV latency. *Nucleic Acids Res.* **45**, 9348–9360 (2017). 28637181 10.1093/nar/gkx550 PMC5766202

[118] Li, H. et al. Lipopolysaccharide-induced methylation of HuR, an mRNA-stabilizing protein, by CARM1. Coactivator-associated arginine methyltransferase. *J. Biol. Chem.* **277**, 44623–44630 (2002). 12237300 10.1074/jbc.M206187200

[119] Liu, J. et al. Arginine methylation-dependent LSD1 stability promotes invasion and metastasis of breast cancer. *EMBO Rep.* **21**, e48597 (2020). 31833203 10.15252/embr.201948597 PMC7001506

[120] Naeem, H. et al. The activity and stability of the transcriptional coactivator p/CIP/SRC-3 are regulated by CARM1-dependent methylation. *Mol. Cell. Biol.* **27**, 120–134 (2007). 17043108 10.1128/MCB.00815-06 PMC1800659

[121] Brook, M. et al. The multifunctional poly(A)-binding protein (PABP) 1 is subject to extensive dynamic post-translational modification, which molecular modelling suggests plays an important role in co-ordinating its activities. *Biochem. J.* **441**, 803–812 (2012). 22004688 10.1042/BJ20111474 PMC3298439

[122] Nie, M. et al. CARM1-mediated methylation of protein arginine methyltransferase 5 represses human gamma-globin gene expression in erythroleukemia cells. *J. Biol. Chem.* **293**, 17454–17463 (2018). 30257864 10.1074/jbc.RA118.004028 PMC6231142

[123] Kim, K. Y. et al. PRMT4-mediated arginine methylation negatively regulates retinoblastoma tumor suppressor protein and promotes E2F-1 dissociation. *Mol. Cell. Biol.* **35**, 238–248 (2015). 25348716 10.1128/MCB.00945-14 PMC4295381

[124] Sims, R. J. et al. The C-terminal domain of RNA polymerase II is modified by site-specific methylation. *Science* **332**, 99–103 (2011). 21454787 10.1126/science.1202663 PMC3773223

[125] Zhao, H., Zhang, Y., Dai, H., Zhang, Y. & Shen, Y. CARM1 mediates modulation of Sox2. *PLoS ONE* **6**, e27026 (2011). 22046437 10.1371/journal.pone.0027026 PMC3203945

